# Induction of ferroptosis in human nasopharyngeal cancer cells by cucurbitacin B: molecular mechanism and therapeutic potential

**DOI:** 10.1038/s41419-021-03516-y

**Published:** 2021-03-04

**Authors:** Shuai Huang, Bihui Cao, Jinling Zhang, Yunfei Feng, Lu Wang, Xiaopei Chen, Hang Su, Shengrong Liao, Jinggong Liu, Jun Yan, Baoxia Liang

**Affiliations:** 1 Department of Orthopaedic Surgery, The Second Affiliated Hospital of Guangzhou Medical University, Guangzhou, 510260 China; 2 Department of Radiology, The Second Affiliated Hospital of Guangzhou Medical University, Guangzhou, 510260 China; 3 Translational Medicine Centre, The Second Affiliated Hospital of Guangzhou Medical University, Guangzhou, 510260 China; 4 CAS Key Laboratory of Tropical Marine Bio-resources and Ecology, Guang dong Key Laboratory of Marine Materia Medica, Research Center for Marine Microbes, South China Sea Institute of Oceanology, Chinese Academy of Sciences, Guangzhou, 510301 China; 5 Guangdong Provincial Hospital of Traditional Chinese Medicine, The Second Affiliated Hospital of Guangzhou University of Chinese Medicine, Guangzhou, 510120 China; 6 Department of Laboratory Medicine, The Second Affiliated Hospital of Guangzhou University of Chinese Medicine, Guangzhou, 510120 China

**Keywords:** Cancer metabolism, Drug development

## Abstract

Cucurbitacin B (CuB) is a widely available triterpenoid molecule that exhibits various biological activities. Previous studies on the anti-tumour mechanism of CuB have mostly focused on cell apoptosis, and research on the ferroptosis-inducing effect has rarely been reported. Herein, we first discovered the excellent cytotoxicity of CuB towards human nasopharyngeal carcinoma cells and elucidated its potential ferroptosis-inducing mechanisms. Morphology alterations of mitochondrial ultrastructure, as observed via transmission electron microscopy, showed that CuB-treated cells undergo ferroptosis. CuB caused intracellular accumulation of iron ions and depletion of glutathione. Detailed molecular mechanism investigation confirmed that CuB both induced widespread lipid peroxidation and downregulated the expression of GPX4, ultimately initiating a multipronged mechanism of ferroptosis. Furthermore, CuB exhibited anti-tumour effects in vitro by inhibiting cellular microtubule polymerization, arresting cell cycle and suppressing migration and invasion. Finally, CuB significantly inhibited tumour progression without causing obvious side effects in vivo. Altogether, our study highlighted the therapeutic potential of CuB as a ferroptosis-inducing agent for nasopharyngeal cancer, and it provided valuable insights for developing effective anti-tumour agents with novel molecular mechanisms derived from natural products.

## Introduction

**Fig. 1:**
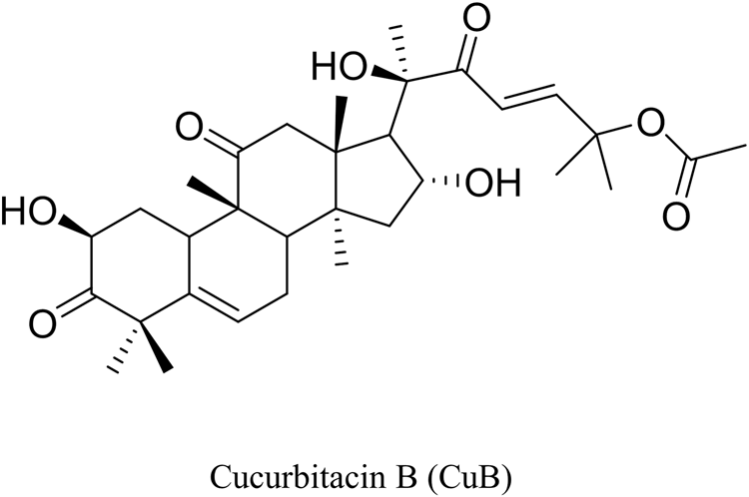
Chemical structure of CuB.

For decades, natural products have been the main source of biologically active substances, especially anti-tumour lead compounds with minimal side effects. The cucurbitacins are a group of tetracyclic triterpenoid natural products derived from oriental herbs that show anti-tumour activity against various human cancers. Cucurbitacin B (CuB, Fig. 1), isolated from Trichosanthes kirilowii Maximowicz, was one of the most abundant and widely studied cucurbitacins derivative^1^. In traditional medicine, CuB has demonstrated strong antifungal, antibacterial, antipyretic, anti-inflammatory, and anticancer activities through regulating multiple signalling pathways^2–6^. It was reported that CuB exhibited potent anti-tumour activity against various tumour cell lines, including liver, breast, and pancreas cancer cell lines^2,7,8^, through inducing cell apoptosis and related pathways. However, some other alternative cell death mechanisms are not fully elucidated.

Canonical caspase-dependent apoptosis has long been recognized as the major mechanism of anti-tumour natural products. However, an increasing number of other novel mechanisms of cell death have recently been identified. Among them, ferroptosis is a newly identified iron-dependent programme of cell death (PCD), which is distinct from the well-studied PCDs such as apoptosis, necrosis, and autophagy^9,10^. Ferroptosis, which is suppressed by iron chelators or lipophilic antioxidants, is characterized by intracellular accumulation of lipid peroxides^11^. Accumulating studies have implicated that dysfunctional ferroptosis is involved in the progression of numerous human diseases and pathogenic processes^12^, including carcinogenesis, ischaemia-reperfusion injury, traumatic spinal cord injury, and neurodegenerative diseases. Since the term ferroptosis was defined by Dr. Brent R. Stockwell in 2012^13^, many studies have demonstrated that ferroptosis is triggered by small molecules, such as the system Xc- inhibitor, erastin, and the glutathione peroxidase 4 (GPX4) inhibitor, (1S,3R)-RSL3^14,15^. In addition, a ferroptosis inducer may enhance the chemosensitivity of drug-resistant cancer cells towards chemotherapeutic drugs^16^. Ferroptosis-based cancer therapies are expected to overcome the limitations of current traditional therapeutics due to resistance to apoptosis or necrosis^17^. Recently, novel anticancer drugs based on the potential therapeutic opportunities and non-apoptotic features of ferroptosis are being developed. These studies have advanced the exploitation of novel ferroptosis inducers as a valid approach in the development of antineoplastic drugs.

In this study, first, we investigated the cytotoxicity effect of CuB on different human tumour cell lines. These initial screening results indicated that the human nasopharyngeal carcinoma CNE1 cell line was most sensitive to CuB. Nasopharyngeal carcinoma was the most common malignant tumour of the nasopharynx and endemic in southern China^18,19^. However, few investigations on the underlying mechanisms of CuB towards this type of cancer were reported. Thus, the CNE1 cell line was selected to investigate underlying the inhibition cell growth mechanism of CuB. Herein, we presented evidence that CuB induced ferroptosis by increasing lipid peroxidation and reducing the expression of GPX4. In addition, the effects of CuB on cytoskeletal damage, cell cycle, and anti-metastatic activity in CNE1 cells were also investigated. Moreover, a nude-xenograft model was established to evaluate the potent anti-tumour activity of CuB in vivo. In summary, these detailed mechanism studies validated the ferroptosis-inducing effect of CuB, verifying a novel non-apoptosis cell death in nasopharyngeal cancer cells. More importantly, the in vitro and in vivo anti-tumour effect of CuB indicated the potential of CuB as a promising naturally derived ferroptosis-inducing agent and ferroptosis-based cancer therapy.

## Materials and methods

### Cell lines and reagents

Human breast carcinoma MCF-7, human ovarian carcinoma A2780, human nasopharyngeal carcinoma CNE1, human liver carcinoma HepG2, human lung carcinoma H157, and human colorectal carcinoma HCT-8 cells were cultured in RPMI 1640 medium supplemented with 10% (v/v) foetal bovine serum (FBS), 100 μg/mL streptomycin and 100 units/mL penicillin in a humidified 5% CO_2_ atmosphere at 37 °C.

### Cell cytotoxicity assays and inhibitor studies

Cell cytotoxicity was measured by a 3-(4, 5-Dimethylthiazol-2-yl)-2, 5-diphenyl tetrazolium bromide (MTT) assay. In brief, CNE1 cells (5000 cells/well) were seeded in 96-well plates and treated with different concentrations of CuB (J&K Scientific Ltd., Beijing, China) with or without Z-VAD-FMK(ZVAD,50 μM), necrostatin-1(Nec1, 50 μM), deroxamine (DFO, 100 μM), ciclopirox olamine (CPX, 5 μM) or ferrostatin-1(Fer-1, 5 μM) for 48 h. ZVAD, Nec1, DFO, CPX, and Fer-1 were purchased from Topscience Co., Ltd. (Shanghai, China). Then, 20 μL of MTT (Sigma, USA) at a final concentration of 0.5 mg/mL was added to each well. After 4 h of incubation at 37 °C, culture supernatants were removed, and 150 μL of DMSO was added. Plates were agitated on a shaker in the dark, and the optical density (OD) values at 490 nm were measured using a microplate reader (Synergy H1, BioTek, USA). The viability of cells treated with the compounds was evaluated by calculating the IC_50_ values with GraphPad Pro Prism 5.0 (GraphPad, San Diego, CA).

### Lactate dehydrogenase (LDH) release assay

CNE1 cells (5000 cells/well) were seeded in 96-well plates and treated with CuB (1 nM, 10 nM, and 50 nM) and with or without ZVAD, Nec1, DFO, CPX, or Fer-1for 24 h. Supernatants from the treated cells were collected, and LDH release was measured at a wavelength of 490 nm using a LDH cytotoxicity assay kit (Thermo Scientific, USA) according to the manufacturer’s instructions.

### Apoptosis analysis

CNE1 cells (1.5 × 10^5^ cells/well) were seeded in a 6-well plate and treated with CuB (1, 10, 50, 200, 500, and 1000 nM) for 48 h. Cells were then harvested and washed twice with PBS. Cells were incubated with FITC-conjugated annexin V and propidium iodide (PI) for 30 min, and the stained apoptotic cells were counted via flow cytometry (Navios, Beckman Coulter, Inc., USA). A minimum of 10,000 cells were harvested for analysis per condition.

### Determination of iron concentration

CNE1 cells (1.5 × 10^5^ cells/well) were seeded in a 6-well plate and treated with CuB (1, 10, and 50 nM) for 24 h. Cells were then washed and lysed. The iron concentration was determined with an iron assay kit (Sigma, USA) according to the manufacturer’s instructions.

### GSH quantification analysis

After CNE1 cells (1.5 × 10^6^ cells/well) were treated with CuB (1, 10, and 50 nM) for 6, 12, and 24 h, GSH levels were measured using a GSH assay kit (Beyotime, Jiangsu, China) following the manufacturer’s instructions. The absorbance at 450 nm was measured in a microplate reader (Synergy H1, BioTek, USA). A standard curve for the GSH concentration was generated along with sample data and was used to calculate GSH levels.

### Analysis of lipid peroxidation

After CNE1 cells (1.5 × 10^6^ cells/well) were treated with CuB (1, 10, and 50 nM) for 24 h, they were harvested by trypsinization and resuspended in 500 μL of PBS containing the C^11^-BODIPY581/591 (Thermo Scientific, USA) probe (2.5 μM). Cells were then incubated for 30 min at 37 °C and analysed using a flow cytometer (Navios, Beckman Coulter, Inc.) equipped with a 488 nm laser for excitation. To determine whether ferroptosis was involved, cells were co-treated with DFO, CPX, Fer-1. A minimum of 10,000 cells were harvested for analysis per condition.

### Transmission electron microscopy (TEM) measurements of cellular ultrastructural morphological changes

CNE1 cells (2 × 10^6^ cells/well) were plated in 60-mm culture dishes. Cells were then treated with CuB (50 nM) and erastin (10 μM) for 24 h. Cells were digested and fixed with 2% glutaraldehyde and 1% osmium tetroxide. Cell samples were then cut into ultrathin sections, stained with 2% uranyl acetate, dehydrated, embedded, and stained with lead citrate. Images were acquired with a transmission electron microscope (HT7700, Hitachi, Ibaraki, Japan).

### Cell cycle analysis

Cell cycle analysis was performed via flow cytometry. CNE1 cells (1.5 × 10^6^ cells/well) were treated with increasing concentrations of CuB (10, 50, and 100 nM) for 24 h. Cells were harvested and washed with PBS, and cells were then fixed with 75% ice-cold ethanol at 4 °C overnight. Cells were then treated with RNase A, stained with 100 mg/mL PI, and subjected to flow cytometric analysis (Navios, Beckman Coulter, Inc., USA). A minimum of 10,000 cells were harvested for analysis per condition.

### Western blot analysis

Western blot assays were performed as described earlier^20^. After cells were treated with different concentrations of CuB (1–1000 nM) for 24 or 48 h, they were lysed on ice in RIPA lysis buffer. The lysate was sonicated and centrifuged for 15 min, and the supernatant was collected. Protein concentrations were determined using a BCA kit, and lysate samples containing 50 μg of protein were subjected to SDS-PAGE. The separated proteins were transferred to PVDF membranes, which were blocked with 5% milk in Tris-buffered saline containing Tween-20 (TBST) for 1.5 h followed by incubation with the indicated primary antibodies overnight at 4 °C. Membranes were washed and further incubated with HRP-conjugated secondary antibodies for 1.5 h at room temperature. Signals were detected with an enhanced chemiluminescence kit (Thermo Scientific, USA). Actin or tubulin was used as the loading control. Antibodies, including anti-caspase 3 (cat.no.#14220), anti-cleaved caspase 3 (cat.no.#9664), anti-PARP (cat.no.#9542), anti-cleaved PARP (cat.no.#5625), anti-bad (cat.no.#9292), anti-bcl-xl (cat.no.#2762), anti-bax (cat.no.#2774), anti-bal-2 (cat.no.#15071),anti-caspase 7 (cat.no.#12827), anti-cleaved caspase 7 (cat.no.#8438), anti-caspase 9 (cat.no.#9508), anti-cleaved caspase 9 (cat.no.#52873), anti-cyclin B1 (cat.no.#4138), anti-cdc25C (cat.no.#4688), anti-cdc2 (cat.no.#77055), anti-p-cdc2 (cat.no.#9116), anti-slug (cat.no.#9585), anti-β-catenin (cat.no.#8480), anti-E-cadherin (cat.no.#3195), anti-N-cadherin (cat.no.#13116), anti-snail (cat.no.#3879), anti-vimentin (cat.no.#5741), anti-GAPDH (cat.no.#5174), anti-tubulin (cat.no.#2148), anti-actin (cat.no.#3700), anti-rabbit IgG-HRP (cat.no.#7074), and anti-mouse IgG-HRP (cat.no.#7076), were purchased from Cell Signaling Technology, Inc. (USA). Anti-GPX4 antibody (cat.no.ab125066) was purchased from Abcam Inc. (USA).

### Determination of GPX4 mRNA expression

After CNE1 cells treatment with CuB (1,10,50 nM) for 24 h, total RNAs were extracted using Trizol reagent (Invitrogen, USA) and dissolved in nuclease-free water. 1 µg RNA was used to reverse transcribe cDNA using Reverse Transcription system Kit (Nanjing Vazyme Biotech Co., Ltd., China) as described in the manufactures’ protocol. cDNA was used for quantitative Real-Time PCR on the QuantStudio 7 Flex (ThermoFisher, MA) using GPX4(fwd: 5′-ACAAGAACGGCTGCGTGGTGAA-3′; rev: 5′-GCCACACACTTGTGGAGCTAGA-3′) or actin (fwd: 5′-ATCTGGCACCACACCTTCTAC-3′; rev: 5′-CAGGTCCAGACGCAGGATG-3′) primers in a SYBR green reaction to determine mRNA levels. GPX4 mRNA expression levels were normalized to the expression of actin mRNA.

### Immunofluorescence microscopy

The effects of CuB on the cell cytoskeleton were assessed with a Cellomics cytoskeletal rearrangement kit according to our previous method^21^. CNE1 cells (1 × 10^5^ cells/dish) were seeded in a confocal culture dish. After treatment with CuB (50 nM) for 24 h, cells were placed on ice for 1 h to depolymerize microtubules. Cells were then pre-incubated in a 37 °C incubator for 0, 3, 6, 9, and 15 min to observe microtubule reassembly. Cells were fixed in 4% paraformaldehyde, permeabilized with 0.1% Triton X-100, and blocked with 5% BSA at room temperature for 1 h. Subsequently, cells were incubated with an anti-tubulin antibody for 2 h. After washing with TBST, cells were incubated with a DyLight® 488-conjugated secondary antibody and Hoechst 33342 in the dark. Fluorescence images were acquired with a confocal laser scanning microscope (Leica Microsystems, Wetzlar, Germany).

### Transwell migration assay

Transwell migration assays were performed with CNE1 cells using a transwell membrane (8 μm pore size, Corning). CNE1 cells (1 × 10^5^ cells/well) were suspended in a serum-free medium with or without CuB (10, 50, and 100 nM) and were then seeded in the upper compartment of the chamber. The lower compartment of the chamber was filled with a medium containing 10% FBS as a chemoattractant. After 24 h of incubation, cells on the upper surface of the filter were removed with cotton swabs, and cells on the lower surface were fixed with 4% paraformaldehyde and stained with 0.1% crystal violet for 15 min. The migrated cells were imaged and counted under a light microscope (Nikon, Japan).

### Invasion assay

Filter inserts that fit into 24-well invasion chambers were coated with Matrigel (BD Biosciences) for the invasion assay. CNE1 cells (1 × 10^5^ cells/well) were seeded in the upper compartment of the chamber. After incubation with CuB (10, 50, and 100 nM) for 24 h at 37 °C, the filter was fixed with 4% paraformaldehyde and then stained with 0.1% crystal violet for 15 min. After the filter was dried, the invaded cells were imaged and counted under a light microscope (Nikon, Japan).

### Subcutaneous tumour xenograft establishment

The animal experiment was performed in accordance with protocols approved by the Institutional Animal Care and Use Committee of Guangzhou Medical University. BALB/c nude mice (5 weeks old, female, Guangdong Medical Laboratory Animal Centre, China) were used for animal experiments. Approximately 4 million CNE1 cells were injected subcutaneously into the right flank of each mouse. Palpable solid tumours developed within a month after tumour cell inoculation, and mice were randomly allocated to four different groups (five mice /group) as follows: control (PBS) group, CuB treatment groups [0.5 mg/kg (low-dose) group and 1 mg/kg (high-dose) group] and gemcitabine (GEM, 25 mg/kg) group. Mice received intraperitoneal injections of PBS, CuB, and gemcitabine 3 times weekly. The tumour dimensions were measured every three days with callipers, and the following formula was applied to calculate the tumour volume: V = [length × (width)^2^]/2. In addition, the tumour sizes and animal weights were monitored weekly. At the end of the experiment, all mice were sacrificed by cervical dislocation, and tumour masses were harvested and weighed. Blood was collected for further analysis. At the same time, all mouse organs (heart, liver, spleen, lung, and kidney) were harvested, fixed with 4% paraformaldehyde, and stained with haematoxylin-eosin (H&E).

### Statistical analysis

Data were analysed with Prism 5.0 (GraphPad Software Inc., San Diego, CA) and are expressed as the means ± SD. Comparisons were performed using an unpaired Student’s test, and difference between groups were analysed using one-way ANOVA. For all analyses, *P* < 0.05 was considered as statistically significant.

## Results

### CuB induces cell death

We first used a MTT assay to evaluate the ability of CuB to affect the metabolic state of various cancer cells. Compared with the other cancer cell lines, the nasopharyngeal carcinoma cell line CNE1 was most sensitive to CuB (IC_50_ = 16 nM) after exposure for 48 h (Table 1). Hence, CEN1 cell line was chose for the further study.

**Table 1:** Cytotoxicity of CuB towards different cancer cell lines in vitro.

Comd	Human cancer cell lines, IC_50_ (μM)					
	MCF-7	A2780	CNE1	H157	HepG2	HCT-8
CuB	0.52 ± 0.12	0.093 ± 0.02	0.016 ± 0.005	0.21 ± 0.09	1.17 ± 0.38	0.086 ± 0.07

IC_50_ = compound concentration required to inhibit cancer cells growth by 50%.

**Fig. 2:**
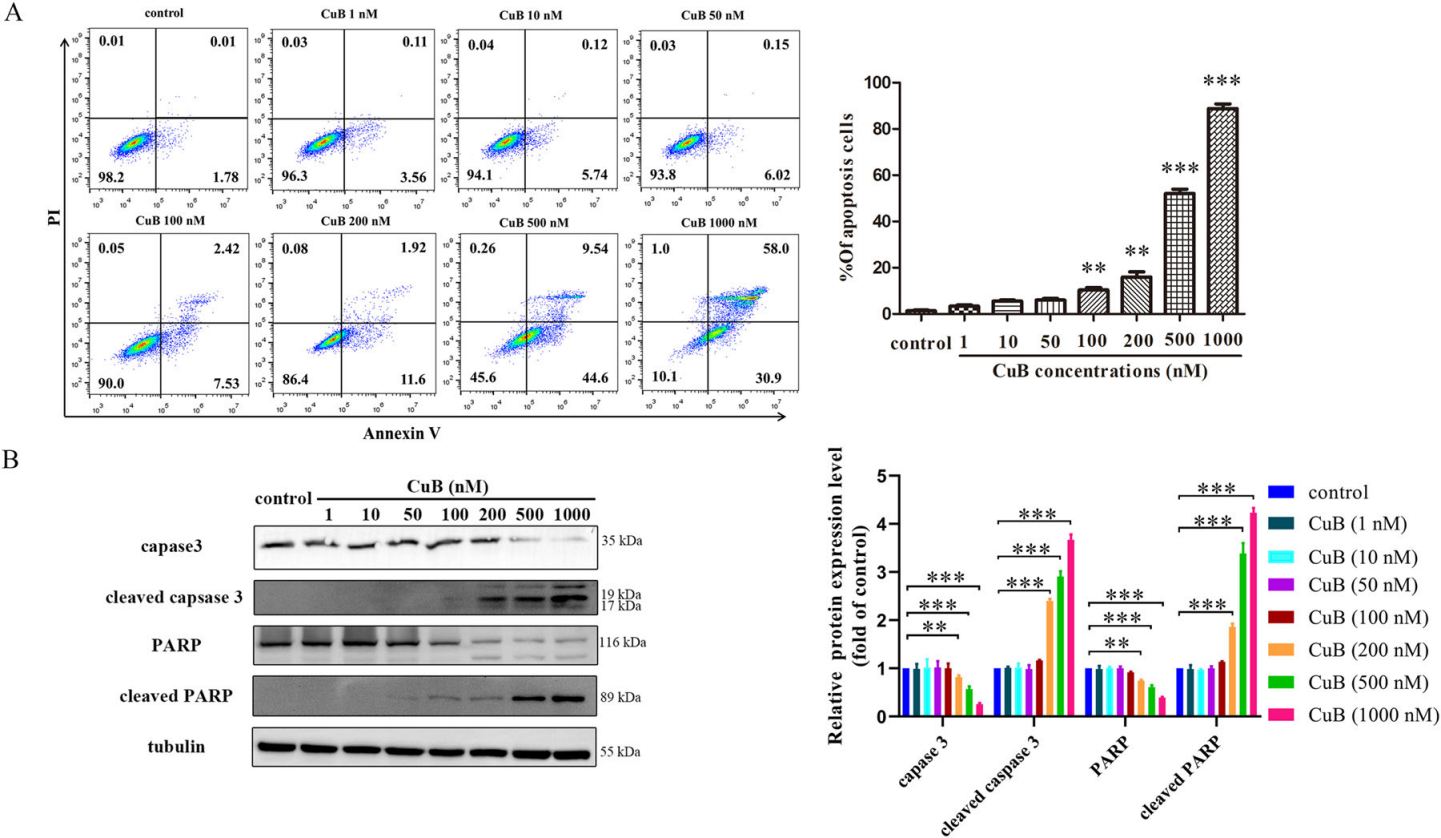
The apoptosis-inducing effect of CuB. CNE1 cells were treated with or without CuB (1, 10, 50, 100,200,500 and 1000 nM) for 48 h. Then, the cells were harvested, stained with Annexin V-FITC/ PI solution for flow cytometer analysis (**A**), or lysed to harvest the total proteins for the western blot analysis of caspase-3, cleaved caspase-3, PARP, and cleaved PARP. ^**^p < 0.01 and ^***^p < 0.001 *vs*. the control group by *t*-test using GraphPad Prism 5.0. **B** A minimum of 10,000 cells were collected for analysis by flow cytometer (Navios, Beckman Coulter). Quantitative analysis was performed by *Image J* (NIH, MD, USA). Data were presented as the mean ± SEM of three independent experiments. ^**^p < 0.01 and ^***^p < 0.001 *vs*. the control group by *t*-test using GraphPad Prism 5.0.

Recent research has shown that CuB and its derivatives induce apoptosis in tumour cells. Thus, an annexin V/PI staining assay was firstly performed to investigate whether cell death occurs through apoptosis in CNE1 cells. Rather unexpectedly, the levels of apoptotic cells increased slightly after CuB treatment below 100 nM of concentration (Fig. 2A). Moreover, the western blot analysis results showed that CuB decreased the expression of caspase 3 and PARP, increased the expression levels of cleaved caspase-3, and cleaved PARP at higher concentrations (200–1000 nM, far more than CuB IC_50_ value) (Fig. 2B). Furthermore, only higher concentrations of CuB (200, 500, 1000 nM) leading to an alteration of the expression levels of apoptosis-related proteins, such as bad, bcx-xl, bax, bcl-2, caspase-7/9, cleaved caspase-7/9. (Supplementary Fig. S1).

**Fig. 3:**
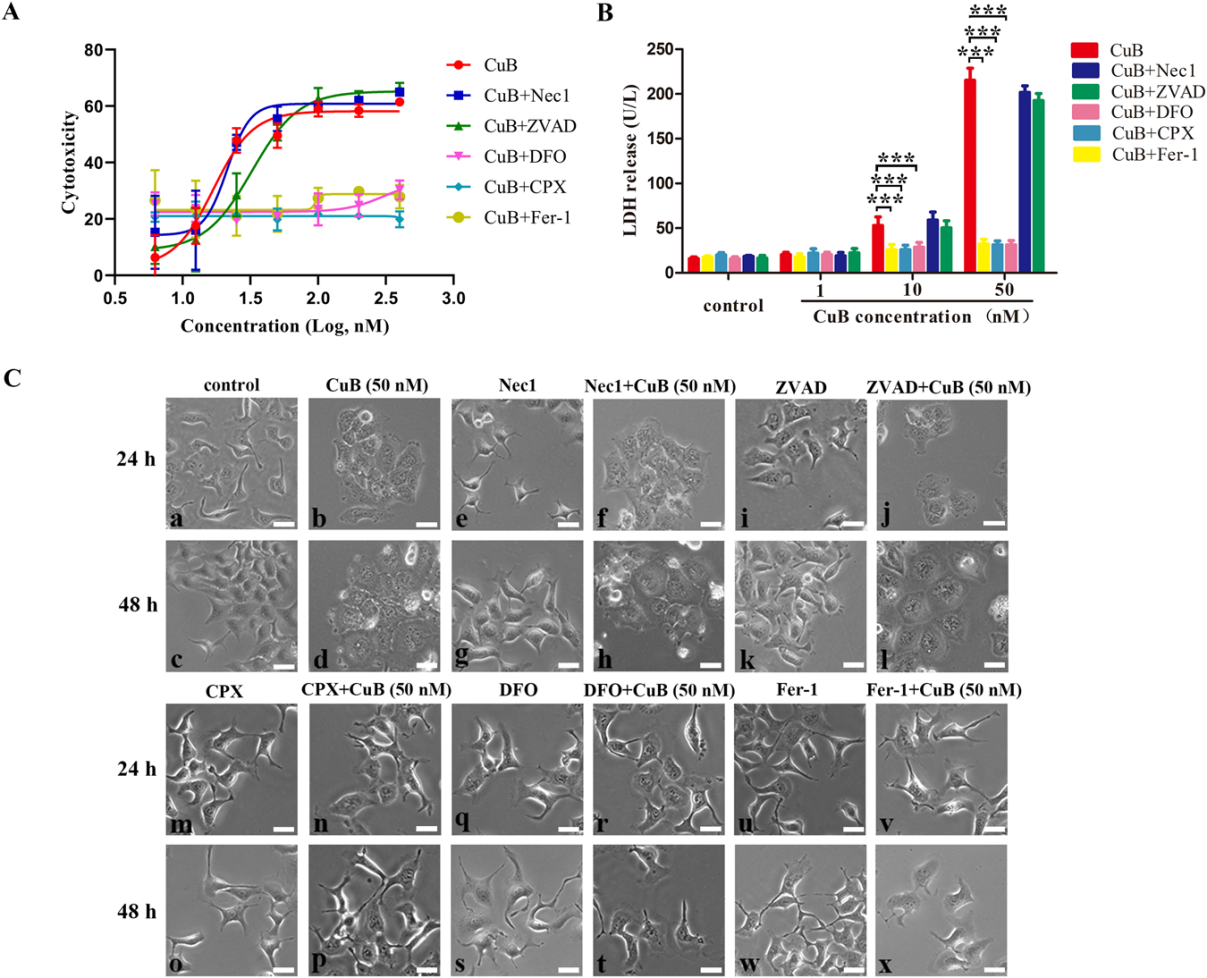
CuB induced cell death of CNE1 cells. **A** The cytotoxicity of CuB at the concentrations of 6.25, 12.5, 25, 50, 100, 200, and 400 nM towards CNE1 cells with or without Nec1 (50 μM), ZVAD (50 μM), Fer-1 (5 μM), CPX (5 μM), or DFO (100 μM) for 48 h, respectively, and the cytotoxicity were determined by MTT assay. **B** The LDH released in CNE1 cells exposed to CuB at the concentrations of 1, 10, and 50 nM with or without Nec1(50 μM), ZVAD(50 μM), Fer-1(5 μM), CPX (5 μM), or DFO (100 μM) for 24 h, respectively. ***p < 0.001 *vs*. the CuB-treated group by t-test using GraphPad Prism 5.0. **C** Representative phase-contrast images of the CNE1 cells exposed to CuB at 50 nM for 24 h (**b**) and 48 h (**d**) with or without Nec1(50 μM, **e**–**h**), ZVAD(50 μM, **i**–**l**), CPX (5 μM, **m**–**p**), DFO (100 μM, **q**–**t**) or Fer-1(5 μM, **u**–**x**) by electron microscope (Nikon, Japan). Scar bar: 20 μm. Data were presented as the mean ± SEM of three independent experiments.

Since it can be concluded from the above results (Table 1 and Fig. 2) that the concentration that induced apoptosis was far exceeded the IC_50_ value of CuB, we have the reason to hypothesize that there might be other forms of cell death induced by CuB. Hence, to define the mode of cell death, the pan-caspase inhibitor (ZVAD), the necrosis inhibitor (Nec1), or the ferroptosis inhibitors (DFO, CPX, and Fer-1) was pre-treated to determine whether these inhibitors neutralize the cytotoxicity of CuB towards CNE1 cells, respectively. As shown in Fig. 3A, only the ferroptosis inhibitors effectively prevent CuB-induced cell death. The fact that DFO, a reported ferroptosis-inhibitor that has iron ion-chelating ability^22^, rescued the cytotoxicity of CuB indicating CuB-induced cell death is iron-dependent. The results of Fig. 3B also validated the significant effect of LDH release induced by CuB (50 nM) and the neutralization effect of ferroptosis inhibitors (Fig. 3B). The cell morphological study demonstrated CuB (50 nM) induced notable morphological alterations such as a distinct ballooning feature and cellular swelling, but lack of classical apoptosis characteristics such as cell shrinking, rounding, and chromatin condensation (Fig. 3C). These morphological alterations were consistent with the reported typical ferroptosis characteristics. Likewise, these morphological alterations were obviously reversed in response to ferroptosis inhibitors, but no corresponding response was observed to apoptosis inhibitor or necrosis inhibitor (Fig. 3C). Collectively, CuB might triggered the cell death of CNE1 cells in the form of ferroptosis and eventually lead to cell death.

### CuB induces ferroptosis in CNE1 cells

**Fig. 4:**
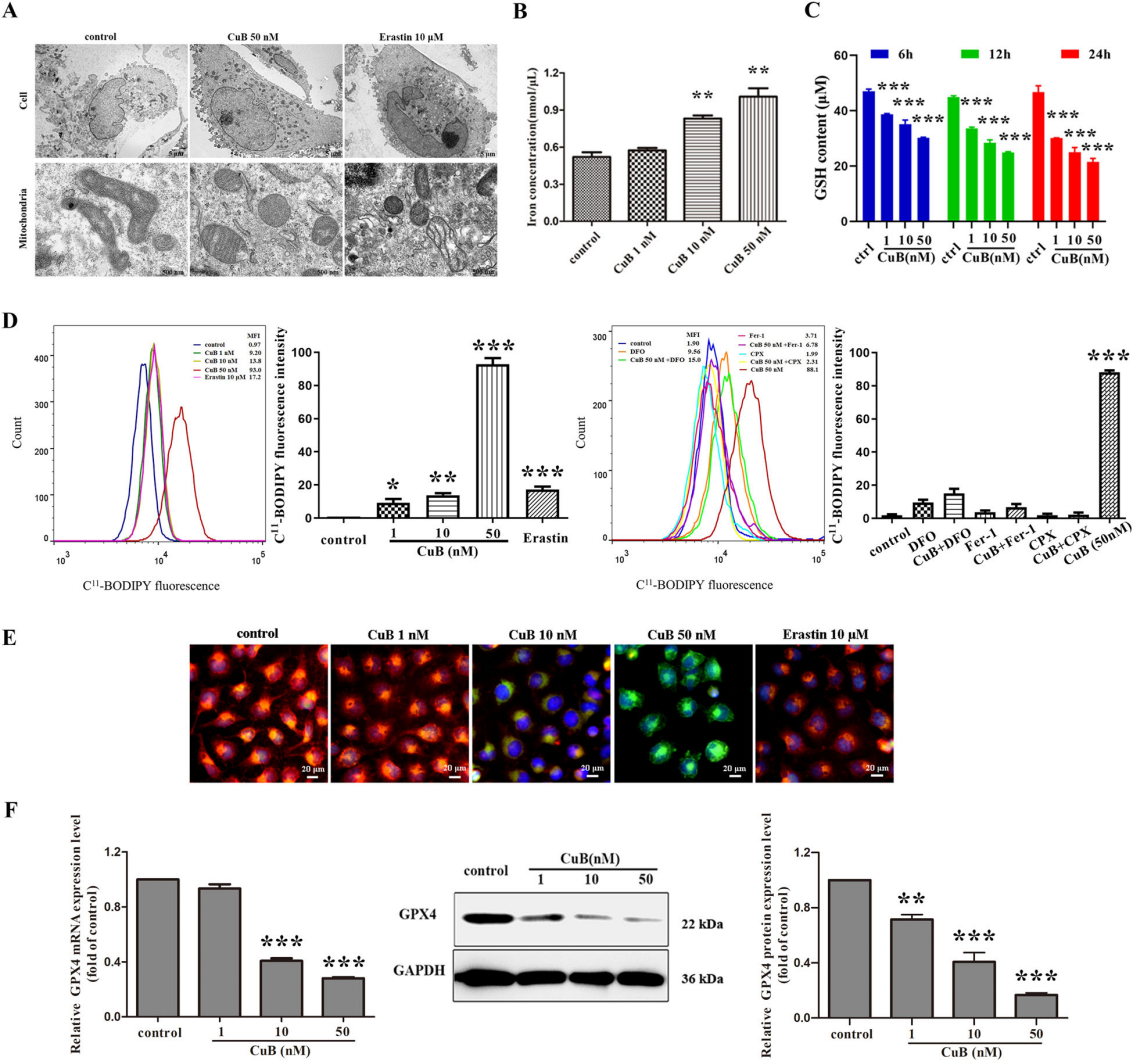
CuB induced ferroptosis. **A** Representative cell and mitochondrial ultrastructural images of CNE1 cells treated with or without CuB (50 nM) or erastin (10 μΜ) for 24 h. The cells were fixed and detected by TEM (HT7700, Hitachi, Ibaraki, Japan). Scale bars: cell, 5 μm; mitochondria, 500 nm. **B** Intracellular iron content in CNE1 cells exposed to CuB (1, 10, and 50 nM) for 24 h was determined with an iron assay kit (Sigma, USA). ^**^p < 0.01 *vs*. the control group by *t*-test using GraphPad Prism 5.0. **C** Intracellular GSH content in CNE1 cells exposed to CuB (1,10 and 50 nM) for 6 h, 12 h, or 24 h was determined with a GSH assay kit (Beyotime, Jiangsu, China). ***p < 0.001 *vs*. the control group by *t*-test using GraphPad Prism 5.0. **D** The lipid peroxidation generation in CNE1 cells exposed to CuB (1, 10 and 50 nM) with or without Nec1(50 μM), ZVAD(50 μM), Fer-1(5 μM), CPX (5 μM), or DFO (100 μM) for 24 h. Then harvested cells were stained with C^11^-BODIPY581/591 and analysed by flow cytometer (Navios, Beckman Coulter). A minimum of 10,000 cells were harvested for analysis. [MFI = mean fluorescence intensity]. *p < 0.05, **p < 0.01, and ***p < 0.001 *vs*. control group by *t*-test using GraphPad Prism 5.0. **E** The fluorescent images of lipid peroxidation in CNE1 cells using C^11^-BODIPY-581/591 probe after incubation with CuB (1, 10, and 50 nM) or erastin (10 μM) for 24 h. Scale bar: 20 μm. (**F**) The mRNA level of GPX4 quantified by qRT-PCR and western blot analysis of GPX4 in CNE1 cells with or without CuB (1, 10, and 50 nM) for 24 h. Quantitative analysis was performed by *Image J* (NIH, MD, USA). ***p < 0.001 *vs*. control group by *t*-test using GraphPad Prism 5.0. Data were presented as the mean ± SEM of three independent experiments.

Ferroptosis is morphologically characterized by alterations in mitochondrial morphological features^23^. In order to validate the ferroptosis-inducing effect of CuB towards CNE1, the mitochondrial ultrastructural alterations induced by CuB were investigated by TEM. The TEM results shown in Fig. 4A indicated that after CuB (50 nM) treatment, CNE1 cells exhibited intracellular shrunken mitochondria, increased mitochondrial membrane density, and reduced mitochondrial cristae compared with control cells, which was consistent with previous reports of ferroptosis. Likewise, stimulation with erastin (10 μM) showed similar effects on mitochondrial structure. These morphological alterations suggested that the programmed cell death induced by CuB in CNE1 cells was distinct from classical apoptotic cell death.

Iron, an essential element in the mitochondrial respiratory chain, performs a pivotal function in ferroptosis^24^. Hence, the intracellular iron concentrations were measured. As shown in Fig. 4B, the intracellular iron concentrations increased after treatment with CuB in a dose-dependent manner. The abundance of iron contributes to the susceptibility of cells to ferroptosis. Moreover, CuB-induced cytotoxicity was neutralized by DFO, CPX, and Fer-1 (Fig. 3E). Altogether, our data indicated that CuB triggered the cell death process in CNE1 cells in an iron-dependent manner. A reduced intracellular GSH level leads to excessive oxidative stress and is a critical element of ferroptosis-mediated cell death. Inactivation of cellular GSH-dependent antioxidant promotes the accumulation of lipid peroxide products and further triggers ferroptosis. As shown in Fig. 4B, CuB caused a significant decrease of the contents of GSH at 6 h and sustained for 24 h. This finding suggested that the GSH depletion caused by CuB may promote a decrease in the cellular antioxidant capacity and may be a crucial factor driving ferroptosis.

Lipid hydroperoxides have been considered as the driving force of ferroptosis and is a hallmark of ferroptosis^25^. The fluorescent probe, C^11^-BODIPY 581/591, used as a lipid peroxidation-sensitive dye. Flow cytometric analysis showed that cellular lipid peroxidation was greatly increased in a dose-dependent manner after treatment with CuB (Fig. 4D). Similarly, CuB-induced lipid peroxidation accumulation was also suppressed by DFO, CPX, and Fer-1. In addition, the process of lipid peroxidation in CNE1 cells was visualized by strong green fluorescence intensity in cells treated with CuB (Fig. 4E), implying CuB effectively causes lipid peroxidation.

The selenoprotein, GPX4, has been identified as a pivotal enzyme for reducing hydroperoxides^26^, especially lipid peroxides, at the expense of the oxidation of two GSH molecules. The above results confirmed that CuB decreased GSH levels and induced lipid peroxidation. To further evaluate the ferroptosis-triggering effect of CuB, the impact of CuB on intercellular GPX4 expression was also investigated. As shown in Fig. S3A, B, CuB almost exerted no effect on the expression of GPX4 after 6 h and 12 h exposure. While, a marked and dose-dependent decrease in the transcriptional levels and protein expression of GPX4 were observed in CNE1 cells after 24 h treatment of CuB (Fig. 4F), and this downregulated effect could be neutralized by DFO (Supplementary Fig. S3C), which further confirmed the potential ferroptosis-inducing effect of CuB in CNE1 cells. Additionally, CuB was unable to inhibit GPX4 enzymatic activity even at very high concentrations (12.5–100 μM) in vitro (Supplementary Fig. S4). In summary, all of the above results indicated that CuB might first trigger intracellular GSH depletion, subsequently down-regulated expression of GPX4, and eventually lead to cell ferroptosis.

### CuB rapidly induces cytoskeletal disruption in vitro

**Fig. 5:**
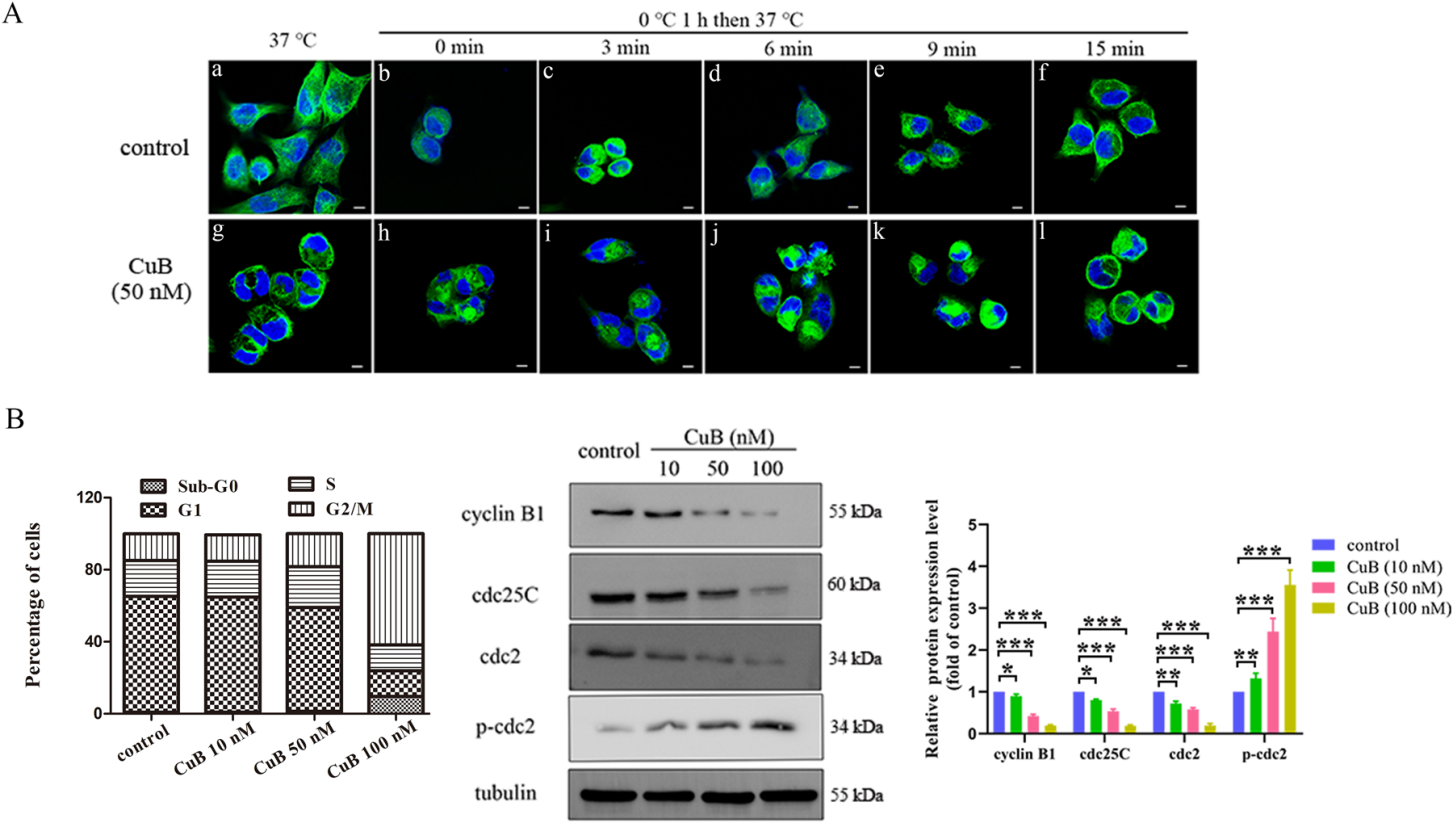
Effects of CuB on cytoskeletal disruption and cell cycle arrest. **A** Representative images of intracellular microtubule networks of CNE1 cells treated with or without CuB (50 nM) 24 h, and then direct detected (**a** and **g**) by confocal laser scanning microscope (Leica Microsystems, Germany). In the microtubules reassembly assay, the untreated or treated cells were placed on ice for 1 h, subsequently warm at 37 °C for 0 (**b** and **h**), 3 (**c** and **i**), 6 (**d** and **j**), 9 (**e** and **k**) and 15 min (**f** and **l**) and then observed by confocal microscopy. The microtubules were stained with β-tubulin mouse antibody and Dylight™ 488-Conjugated Goat Anti-Mouse IgG secondary antibody. The nucleus was stained by Hoechst33342. Scale bars: 10 μm. (**B-C**) The effect of CuB on cell cycle arrest in CNE1 cells. CNE1 cells were treated with or without CuB at 10, 50, and 100 nM for 24 h, and then harvested cells were analysed for PI-stained DNA content by flow cytometry (**B**) or lysed for western blot analysis of cyclinB1, cdc25C, cdc2, and p-cdc2 (**C**). A minimum of 10,000 cells were harvested and analysed by EXPO32 ADC software. Quantitative analysis of western blots was performed by *Image J* (NIH, MD, USA). ^*^p < 0.05, ^**^p < 0.01, and ^***^p < 0.001 *vs*. control group by *t*-test using GraphPad Prism 5.0. Data were presented as the mean ± SEM of three independent experiments.

To gain further insight into the anti-tumour effects of CuB, we evaluated damage to the cell cytoskeleton upon CuB treatment. After cells were incubated with CuB (50 nM) for 24 h, the cytoskeletal network was visualized by confocal laser scanning microscopy. CuB caused significant damage to the cytoskeleton of CNE1 cells. To further investigate the effect of CuB on microtubule growth, we assessed microtubule reassembly after cold-induced depolymerization to characterize the dynamics of intracellular microtubule regrowth. As shown in Fig. 5A, the microtubules in control cells repolymerized during the warm-up process and established a proper microtubule network within 15 min. In contrast, microtubules did not reassemble during interphase in cells treated with CuB. These results indicated that CuB inhibits microtubule assembly, thereby leading to disruption of intracellular microtubules.

In view of the superior anti-proliferative activity of CuB towards cancer cell lines, and in vitro tubulin polymerization inhibitory assay was also performed. The tendency of tubulin aggregation was not inhibited even when cells were incubated with high concentration of CuB (50 µM) (Supplementary Fig. S2). These results further confirmed that exposure of CNE1 cells to CuB destroys the cellular cytoskeleton and it’s accompanied by the occurrence of ferroptosis.

### CuB induces cell cycle arrest in CNE1 cells

Considering the important regulatory function of microtubules in cell mitosis, the cellular DNA content at different phases of the cell cycle was evaluated after CuB treatment. After treatment with 100 nM CuB for 24 h, the percentage of cells in G2/M phase was increased by approximately four-fold (Fig. 5B). The cell cycle was arrested at G2/M phase in CuB-treated cells with a concomitant decrease in the percentage of cells in G1 and S phases. To further elucidate the functional effects of CuB on the cell cycle, the expression of cell cycle-related proteins was evaluated via western blotting. Treatment with CuB significantly decreased the levels of cyclinB1, cdc2, and cdc25C but increased the levels of p-cdc2 in CNE1 cells (Fig. 5C). These results indicated that CuB induces cell cycle arrest at G2/M phase.

### CuB inhibits the migration and invasion of CNE1 cells

**Fig. 6:**
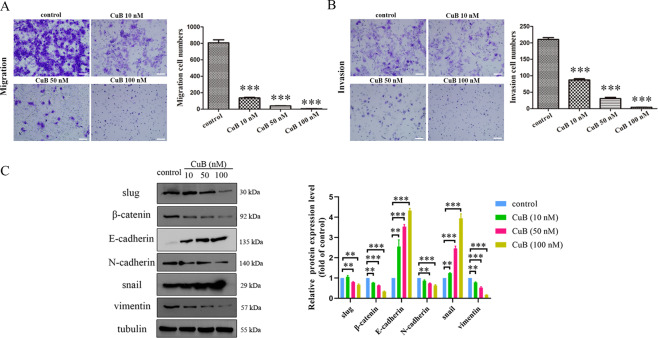
The effects of CuB on migration and invasion in CNE1 cells. **A**, **B** After 24 h of incubation with CuB (10, 50, and 100 nM), migratory and invasive behaviours were analysed using migration and matrigel invasion assays in CNE1 cells. ^***^p < 0.001 *vs*. control group by *t*-test using GraphPad Prism 5.0. Scale bars: 100 μm. **C** Western blot analysis of expressions of slug, β-catenin, E-cadherin, N-cadherin, snail, and vimentin in CNE1 cells with CuB (10, 50, and 100 nM) treatment for 48 h. Quantitative analysis of western blots was performed by *Image J* (NIH, MD, USA). ^**^p < 0.01 and ^***^p < 0.001 *vs*. the control group by *t*-test using GraphPad Prism 5.0. Data were presented as the mean ± SEM of three independent experiments.

Viswanathan and co-workers found that epithelial-mesenchymal transition (EMT) is sensitive to ferroptosis^27^. To determine whether CuB inhibits the migration and invasion of CNE1 cells, transwell assays with or without Matrigel were used. As depicted in Fig. 6A, B, the inhibitory effect of CuB on CNE1 cell migration and invasion was observed. The migration and invasion of CNE1 cells were markedly reduced in a dose-dependent manner. To further investigate the molecular mechanism involved in EMT, the expression of related proteins regulating migration and invasion was assessed by western blotting. As shown in Fig. 6C, CuB significantly downregulated the expression of slug, β-catenin, and N-cadherin but upregulated the expression of E-cadherin and snail in CNE1 cells. These results revealed that CuB exerts an anti-metastatic effect on CNE1 cells.

### CuB inhibits tumour growth in vivo

**Fig. 7:**
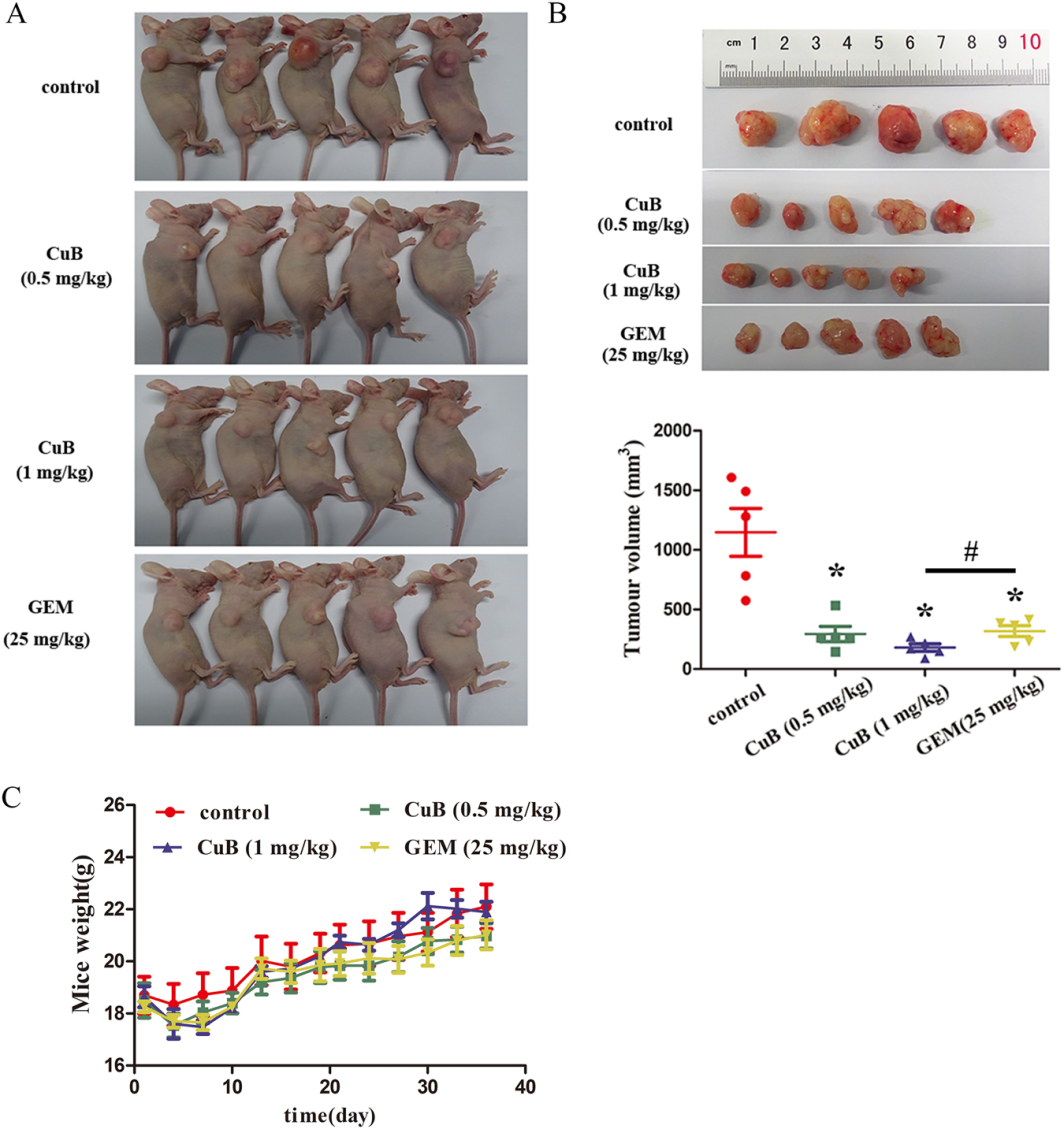
In vivo anti-tumour effect of CuB. **A** Images of euthanized mice with or without the administration of CuB (0.5, 1 mg/kg) and GEM (25 mg/kg). **B** Images of excised tumour bulks and final average tumour volume in each group. **C** Body weight of mice from each group during the whole observation period. Data were presented as the mean ± SEM, ^*^p < 0.05 vs. the control group by *t*-test using GraphPad Prism 5.0, *n* = 5. ^#^ *p* < 0.05 for between group comparisons by one-way ANOVA using GraphPad Prism 5.0, *n* = 5.

Based on the in vitro findings described above, a human nasopharyngeal carcinoma murine xenograft model was established to examine the in vivo anti-tumour effect of CuB in BALB/c mice.CNE1 tumour-bearing mice were intraperitoneally injected with CuB (0.5 mg/kg and 1 mg/kg), gemcitabine (25 mg/kg) or control (PBS) every three days. Gemcitabine, a chemotherapeutic drug commonly used for clinical cancer therapy, was chosen as the positive control. CuB treatment inhibited tumour growth in mice (Fig. 7A, B). At the end of the observation period, the mean tumour volume in the control group was 1.1 ± 0.40 cm^3^ compared to 0.29 ± 0.13 cm^3^ (0.5 mg/kg CuB), 0.18 ± 0.06 cm^3^ (1 mg/kg CuB), and 0.32 ± 0.09 cm^3^ (25 mg/kg GEM) in CuB and gemcitabine-treated mice.

**Fig. 8:**
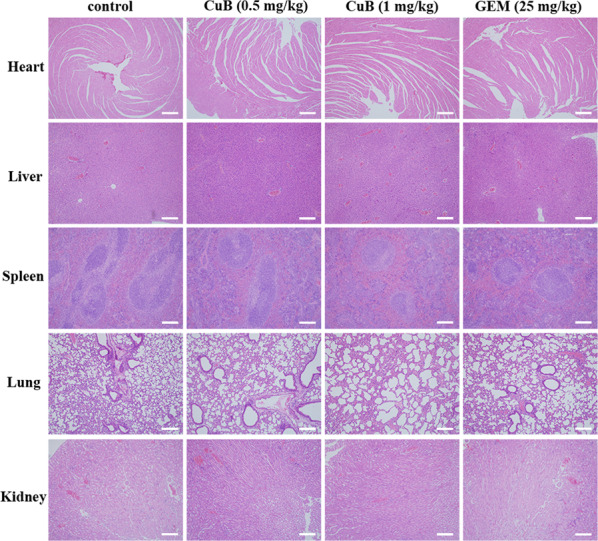
The representative images of H&E staining of heart, liver, spleen, lung, and kidney from mice in each group at the end of the observation period. Scale bars: 100 μm.

To evaluate side effects, changes in the body weights in each group of mice were monitored and recorded. Figure 7C summarizes the body weights. The mean body weight in the control group was 22.1 ± 1.71 g compared to 20.9 ± 0.97 g (0.5 mg/kg CuB group) and 21.9 ± 0.77 g (1 mg/kg CuB group) in CuB-treated mice. The mean bodyweight of the mice in the 25 mg/kg gemcitabine group was 21.4 ± 1.11 g. These data indicated that CuB treatment was well tolerated and has good biocompatibility without resulting in significant body weight loss in mice. In addition, the results in the gemcitabine treatment groups resembled those in the CuB (low dose) treatment group. In contrast, the tumour tissues in the high dose CuB group were significantly smaller than those in mice in the gemcitabine group. These results demonstrated that the high dose of CuB (1 mg/kg) was more effective than the first-line treatment gemcitabine (25 mg/kg) in vivo. Moreover, after CuB administration, haemanalysis showed that CuB had no obvious effect on several blood biochemical parameters (Table 2). Pathomorphological analysis of major organs (including the heart, liver, spleen, lungs, and kidneys) in the four groups after a treatment period of 36 days was performed via H&E staining. No visible signs of toxicity or metastasis in internal organs were observed in all treated mice (Fig. 8). Overall, our findings suggested that CuB exerts anti-tumour effects in vivo and should be further considered for cancer therapy.

**Table 2:** Blood level of different biochemical parameters among study groups.

Group	AST	ALT	BUN	WBC	Hgb	PLT
Normal value	54-298 (U/L)	31-46 (U/L)	0-140 (mg/dL)	1.8-10.7 (K/μL)	110-143 (g/L)	592-2972 (k/μL)
control	151.1 ± 5.5	42.1 ± 0.5	17.9 ± 1.2	4.7 ± 0.9	135.6 ± 1.7	792.6 ± 77.8
CuB (0.5 mg/kg)	183.3 ± 5.4	43.1 ± 4.9	18.3 ± 0.5	5.1 ± 0.9	133.2 ± 4.1	808.2 ± 53.1
CuB (1 mg/kg)	209.5 ± 6.1	50.9 ± 1.7	17.7 ± 0.4	4.6 ± 0.8	131.0 ± 1.9	1097.6 ± 71.5
GEM(25 mg/kg)	175.9 ± 13.5	46.4 ± 3.9	17.1 ± 1.7	4.8 ± 0.8	136.4 ± 1.7	803.2 ± 72.8

*AST* Aspartate aminotransferase, *ALT* Alanine aminotransferase, *BUN* Blood urea nitrogen, *WBC* White blood cells, *Hgb* Haemoglobin, *PLT* Patelets.

## Discussion

Natural products have historically provided the vast majority of small-molecule candidates for evaluation as anticancer agents. Plant-derived agents contain novel unique structures, provide powerful tools for investigating protein function and cell death mechanisms. Among natural cucurbitacins, CuB has been identified as a potential anti-tumourigenic drug due to its pronounced antiproliferative activity. However, the mechanism of its anti-tumour activity has not been thoroughly elucidated. Moreover, to our knowledge, the therapeutic effect of CuB in nasopharyngeal cancer has not been reported. In this study, we provide novel evidence that CuB induces ferroptosis and attempted to investigate the underlying mechanisms. Moreover, the anti-tumour effect of CuB was evaluated in vitro and in vivo.

Previous studies of CuB have focused mainly on its anti-proliferative and inducing apoptosis effects were attributable to suppression of the STAT3 and the Raf/MEK/ERK pathways. Herein, we showed that CuB exhibited marked in vitro cytotoxicity to various tumour cell lines, especially CNE1 cells. However, flow cytometric and western blot analyses of apoptosis-related proteins showed that only higher concentration of CuB (200–1000 nM, far more than of IC_50_ value) induced cell apoptosis, which suggested a mechanism of cell death other than apoptosis. Interestingly, we found that morphology changes in cells were visually different from apotosis. Meanwhile, CuB-induced cell death was prevented by apoptosis and necrosis inhibitors. The same phenomenon in SW480 cell has been observed by Shusuke Yasuda et al.^28^. Furthermore, CuB-induced cytotoxicity was rescued by DFO, CPX, and Fer-1 these ferroptosis inhibitors. Overall, these results provide evidence that the cytotoxicity of CuB to CNE1 cells is iron-dependent. As a further confirmation, we observed the ferroptotic mitochondrial ultrastructural changes upon CuB treatment. Compatible with mitochondrial pivotal role that mitochondria play in ferroptosis^29^, thus, CuB-inducing morphological features implies that cells undergo ferroptosis.

Ferroptosis has recently been identified as a novel mechanism of cell death associated with pathological and physiological processes, and it has recently become a promising therapeutic target for innovative drug development. Accumulating evidence has demonstrated that ferroptosis inducers exhibit an effective anti-tumour activity^30,31^. Iron deposition is a crucial pathological event in ferroptosis and dysregulated iron metabolism can cause ferroptosis^32^. Moreover, excess intracellular iron participates in the Fenton reaction and produces lipid peroxides, resulting in ferroptosis. Our findings revealed that CuB led to an increase of intracellular iron ions concentration, which was advantageous for enhancing oxidative toxicity. Additionally, stable endogenous GSH functions as an important antioxidant to protect cells against oxidative stress and ferroptosis. Obviously, CuB caused intracellular GSH depletion after 6 h treatment, and GSH contents were consecutively declined with the extension of the treatment time, which indicated that CuB could accumulate intracellular thiols sustainably. In this sense, excessive thiols and peroxides disrupt the intracellular redox homeostasis, which further initiates iron-dependent ferroptosis. This finding was similar to that reported in erastin-treated cells where GSH was depleted and cell death was further induced^33^. As a further investigation, CuB significantly induced lipid peroxidation, and this effect was largely reversed by a specific inhibitor (DFO) of ferroptosis, confirming that ferroptosis was caused by overloading lipid peroxidation. Additionally, GPX4 participates as a negative regulator of ferroptosis and a core element in lipid peroxide production during ferroptotic cell death^34^. In contrast to previously reported ferroptosis inducers that target GPX4 by inhibiting its activity^35^, after 24 h exposure, CuB downregulated GPX4 expression, which was the same effect as reported dihydroartemisinin in CNE1 cells^36^. Moreover, the reduction of GPX4 prevents the conversion of allylic lipid hydroperoxides into their corresponding alcohols or free hydrogen peroxide, thereby promoting ferroptosis. In our results, CuB effectively down-regulated GPX4 expression, which attenuated the cellular antioxidant capacity and elevated lipid peroxide levels, thus leading to ferroptosis. Earlier investigations reported that erastin can decrease GSH but not effect GPX4 expression, RSL3 inhibits GPX4 activity but cannot reduce GSH contents. It is worthwhile to point out that, different from this research, our studies demonstrated that under the combined effects of intracellular GSH depletion and down-regulated expression of GPX4 collectively contribute to the ferroptosis-inducing effect of CuB. Considering other current reports in addition to (i) the increase in cellular iron ions levels presented in this study, (ii) the significantly depleted content of GSH, (iii) the induction of lipid peroxidation and downregulation of GPX4 expression, it is reasonable to assert that CuB effectively induce ferroptosis. In summary, we provided the first evidence to demonstrate that CuB induced cell death in a ferroptosis and apoptosis hybrid pathway, in which the predominant mode of cell death in CNE1 cells was ferroptosis.

Studies on the in vitro anti-tumour activity of CuB have reported that it can disrupt the cytoskeletal architecture in several types of cancer cells. However, the effect of CuB on intracellular microtubule reassembly has not been addressed. The cytotoxic effects of CuB may result in morphological changes in the cytoskeleton and modifications of microtubule-mediated functions. We determined that exposure to CuB damaged the microtubule network. In recent years, our group has been committed to the research and development of several anti-tumour drugs derived from natural products that target the tubulin-microtubule system^21,37–42^. We have verified that CuB does not affect purified tubulin polymerization in vitro. CuB does not target tubulin, and the microtubule cytoskeleton disrupted by CuB contributes to ferroptosis induction. Meanwhile, the results obtained in the present work provided a new insight for future research efforts on the association between ferroptosis and microtubule cytoskeleton. Given that disruption of microtubule dynamics generally causes cell growth inhibition and cycle arrest. Here, we observed that treatment with CuB caused cell cycle arrest at G2/M phase. Compatible with CuB destroyed the microtubule network and induced cell cycle arrest, evidence was provided that exposure to CuB resulted in ferroptosis remodelling a disorganization of the cytoskeleton. It has recently been suggested that the occurrence of ferroptosis in cell is conducive to inhibition of cells migration and invasion. In our study, CuB significantly decreased CNE1 cell migration and invasiveness in a dose-dependent manner, which was consistent with those of previous studies showing that CuB inhibits metastasis in breast cancers^43^. This strongly suggests that induction of ferroptosis offers new perspectives on suppressing tumour cells migration and invasion, also providing a potential approach for preventing nasopharynx cancer metastasis.

In vitro, CuB (1 mg/kg) markedly inhibits tumour growth and had a higher efficiency than gemcitabine. Despite its cytotoxicity in vitro, CuB had no significant impact on mouse body weights, and mice showed good tolerance to therapy without obvious signs of toxicity. These results were consistent with those observed for other cucurbitacins analogues^44,45^, further demonstrating its promising advantage as an effective eradication of malignant tumours. Of course, this future study can be the establishment of the ferroptosis relevant animal model to evaluate the basic parameters to gain insight into the clinical benefits of CuB in vivo.

In conclusion, this study for the first time revealed that CuB induces cell death via the ferroptosis pathway. Furthermore, the detailed mechanism indicated that CuB strongly promotes the accumulation of iron ions and GSH depletion, resulting in the production of excess lipid peroxides. In addition, CuB also downregulates the expression of GPX4, thereby initiating a multipronged mechanism of ferroptosis in CNE1 cells. Moreover, we determined that CuB exhibits tumour inhibition effects in vitro and vivo. Based on these findings, we suggest that CuB has therapeutic application as a promising natural candidate for the development of ferroptosis-inducing agents. CuB can be considered as a promising chemotherapeutic ferroptosis-inducing agent for anti-tumour treatment.

## Supplementary information

Supplementary Figure Legends

Supplementary Figure S1

Supplementary Figure S2

Supplementary Figure S3

Supplementary Figure S4
