# Supplementary material for: Induction of ferroptosis in human nasopharyngeal cancer cells by cucurbitacin B: molecular mechanism and therapeutic potential
Source: Cell Death Dis. 2021 Mar 4;12(3):237. doi: 10.1038/s41419-021-03516-y (PMC7933245; doi:10.1038/s41419-021-03516-y)
Supplement: Supplementary file 1 — Supplementary Figure Legends [file 41419_2021_3516_MOESM1_ESM.docx]

**Figure S1.** Effects of cucurbitacin B on bad,bcl-xl,bax,bcl-2,caspase 7, cleaved caspase 7, caspase 9, and cleaved caspase 9 expressions. CNE1 cells were treated with or without CuB (200,500 and 1000 nM) for 48 h. Then cells lysed to harvest the total proteins for the western blot analysis. Quantitative analysis was performed by *Image J* (NIH, MD, USA). Data were presented as the mean ± SEM of three independent experiments. ^*^*p* < 0.05, ^**^*p* < 0.01 and ^***^*P* < 0.001 *vs.* the control group by *t*-test using GraphPad Prism 5.0.

**Figure S2**. Effects of CuB on purified tubulin polymerization in vitro. CuB (10 ,20 μM) were mixed with purified tubulin in general tubulin buffer containing 1 mM GTP and 20% glycerol. Microtubules polymerization was monitored in the kinetic model (excitation wavelength: 360 nm; emission wavelength: 450 nm).

**Figure S3**. Detect GPX4 expression in CNE1 cells exposure to CuB (1, 10 and 50 nM). （A-B）After CuB treatment for 6 h or 12 h (C) After DFO(100 μM) and CuB co-treatment for 24 h, cells were lysed to harvest the total proteins for the western blot analysis. Quantitative analysis was performed by *Image J* (NIH, MD, USA). *p <0.05, **p < 0.01 vs. the control group by t-test using GraphPad Prism 5.0. Data were presented as the mean ± SEM of three independent experiments.

**Figure S4**. Effects of CuB on GPX4 enzymatic activity in vitro. CuB(100,50,25,12.5 μM) were incubated with 2 ug GPX4(Cloud-Clone Corp., China) for 5 min at room temperature in 200 μL of 0.1 M Tris-HCl, pH 7.8 containing 2 mM EDTA, 2.5 mM GSH, 0.1% (v/v) Triton X-100, 0.2 mM NADPH/H^+^ and 25 U/ml glutathione reductase(GR, sigma). Enzymatic activity was triggered by adding 25 μM phosphatidylcholine hydroperoxide (PCOOH) and quantified as the decrease of absorbance at 340 nm due to NADPH/H^+^ oxidation by GR. PCOOH was prepared by oxidationof 1,2-dilinoleoyl-3-phosphatidylcholine (PC) with soybean lipoxidase as described previously (John K. Eaton et al., Nature Chemical Biology 2020).
