# Supplementary figures and images for: Induction of ferroptosis in human nasopharyngeal cancer cells by cucurbitacin B: molecular mechanism and therapeutic potential
Source: Cell Death Dis. 2021 Mar 4;12(3):237. doi: 10.1038/s41419-021-03516-y (PMC7933245; doi:10.1038/s41419-021-03516-y)

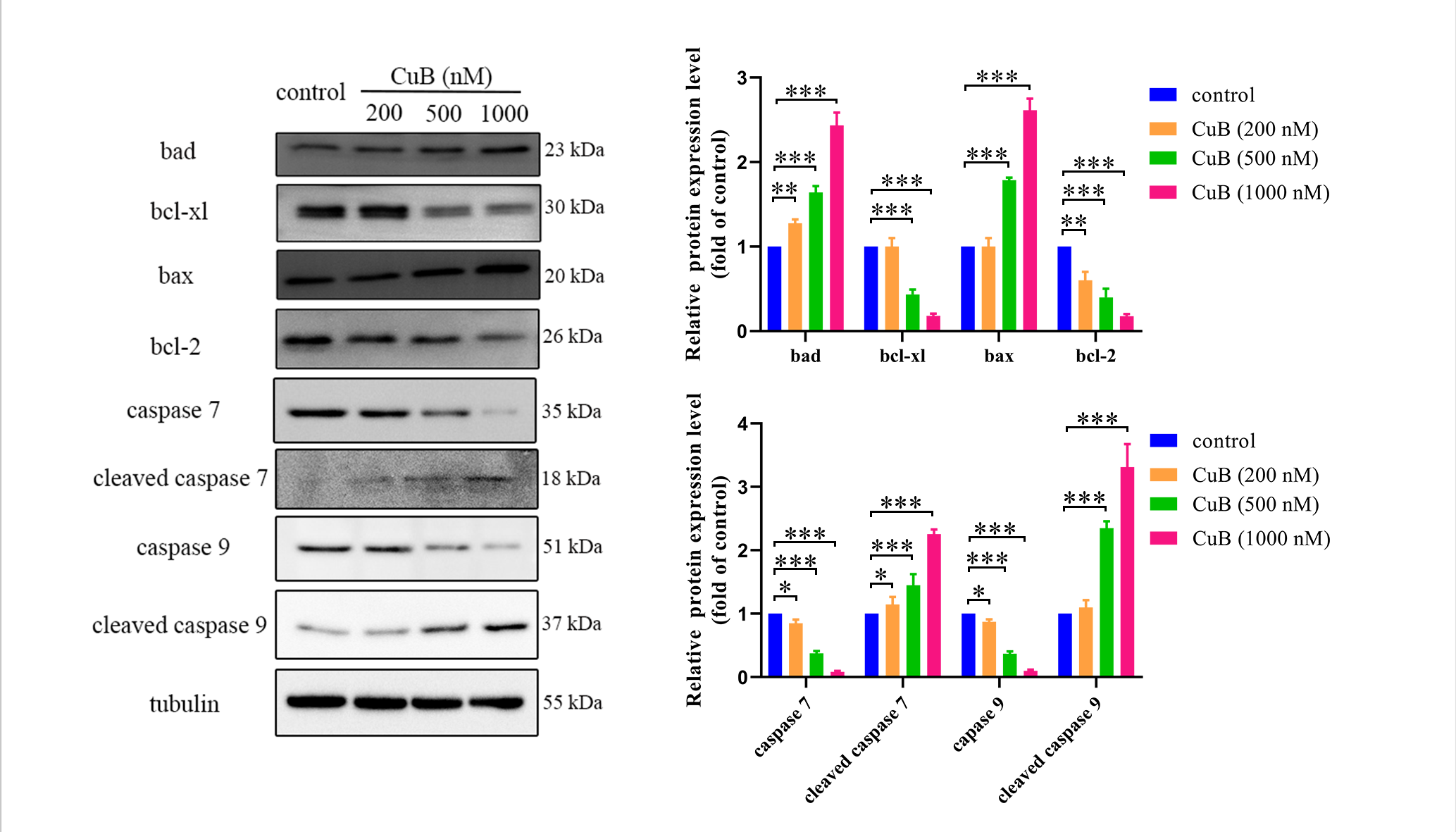

Supplement: Supplementary file 2 — Supplementary Figure S1 [file 41419_2021_3516_MOESM2_ESM.tif]

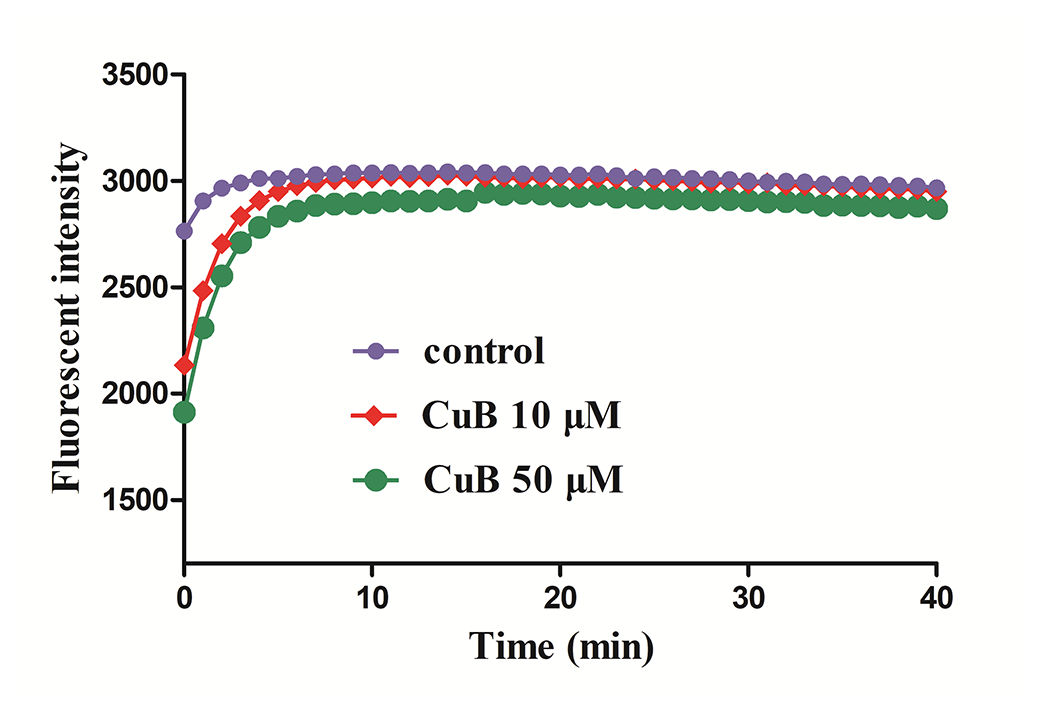

Supplement: Supplementary file 3 — Supplementary Figure S2 [file 41419_2021_3516_MOESM3_ESM.tif]

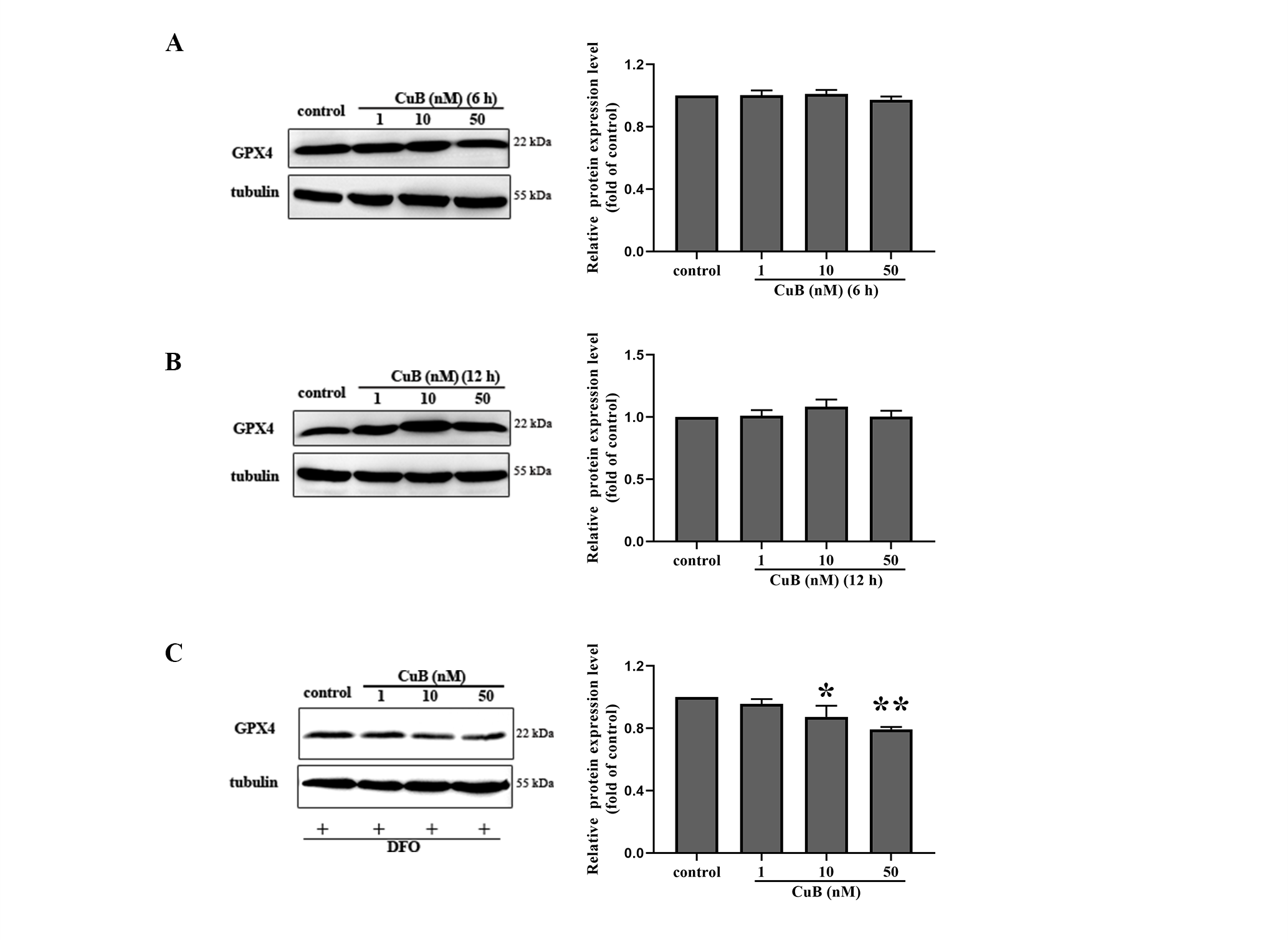

Supplement: Supplementary file 4 — Supplementary Figure S3 [file 41419_2021_3516_MOESM4_ESM.tif]

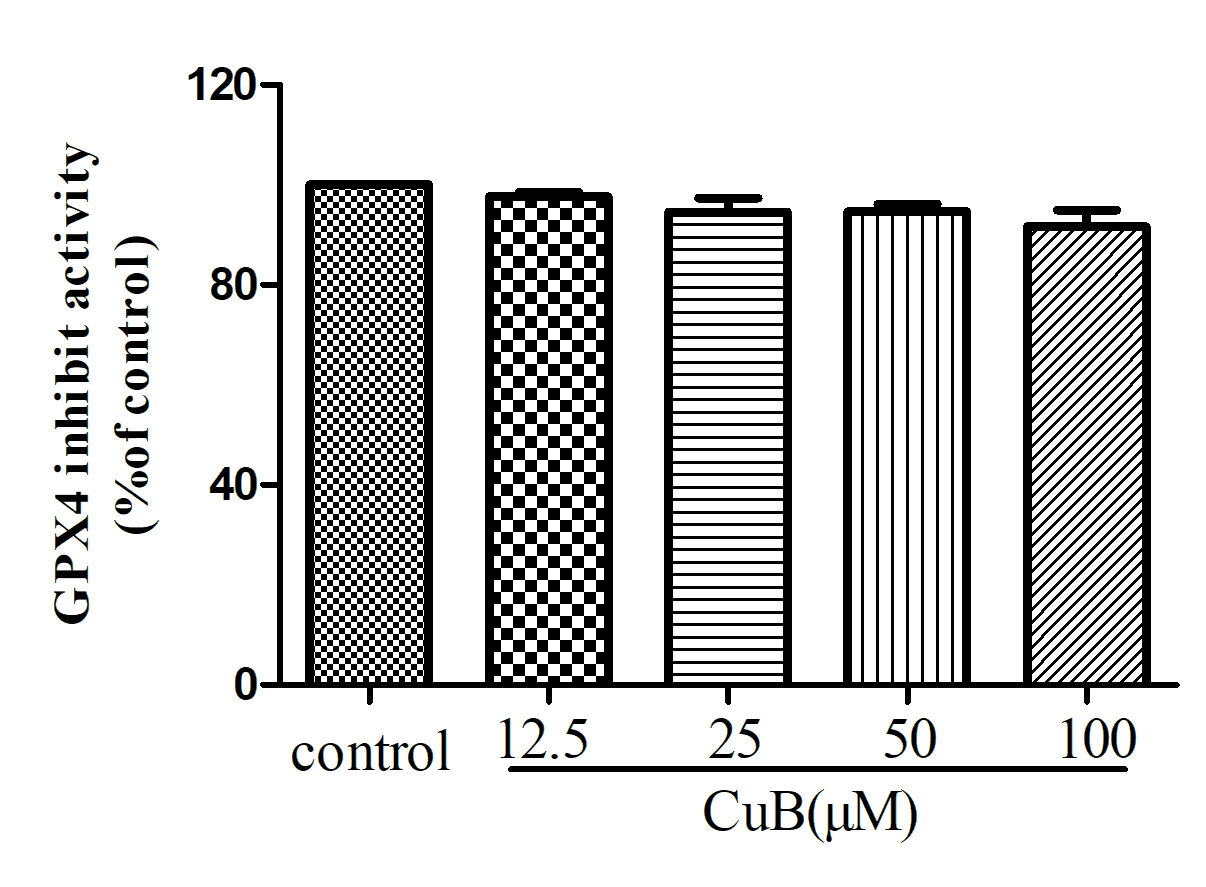

Supplement: Supplementary file 5 — Supplementary Figure S4 [file 41419_2021_3516_MOESM5_ESM.tif]
